# Karyotype Aberrations in Action: The Evolution of Cancer Genomes and the Tumor Microenvironment

**DOI:** 10.3390/genes12040558

**Published:** 2021-04-12

**Authors:** Nicolaas C. Baudoin, Mathew Bloomfield

**Affiliations:** 1 Department of Genetics, The University of Texas MD Anderson Cancer Center, Houston, TX 77030, USA; 2 Department of Biological Sciences and Fralin Life Sciences Institute, Virginia Tech, Blacksburg, VA 24061, USA

**Keywords:** aneuploidy, polyploidy, tetraploidy, cancer, karyotype aberrations, tumor microenvironment, tumor ecology, niche construction, tumor evolution

## Abstract

Cancer is a disease of cellular evolution. For this cellular evolution to take place, a population of cells must contain functional heterogeneity and an assessment of this heterogeneity in the form of natural selection. Cancer cells from advanced malignancies are genomically and functionally very different compared to the healthy cells from which they evolved. Genomic alterations include aneuploidy (numerical and structural changes in chromosome content) and polyploidy (e.g., whole genome doubling), which can have considerable effects on cell physiology and phenotype. Likewise, conditions in the tumor microenvironment are spatially heterogeneous and vastly different than in healthy tissues, resulting in a number of environmental niches that play important roles in driving the evolution of tumor cells. While a number of studies have documented abnormal conditions of the tumor microenvironment and the cellular consequences of aneuploidy and polyploidy, a thorough overview of the interplay between karyotypically abnormal cells and the tissue and tumor microenvironments is not available. Here, we examine the evidence for how this interaction may unfold during tumor evolution. We describe a bidirectional interplay in which aneuploid and polyploid cells alter and shape the microenvironment in which they and their progeny reside; in turn, this microenvironment modulates the rate of genesis for new karyotype aberrations and selects for cells that are most fit under a given condition. We conclude by discussing the importance of this interaction for tumor evolution and the possibility of leveraging our understanding of this interplay for cancer therapy.

## 1. Introduction

Cancer has been widely described as a process of Darwinian evolution. In a manner analogous to speciation, cancer cells genomically and phenotypically diverge into distinct populations (often referred to as clones or stem-lines) that coexist in the same tumor [1]. This heterogeneity is further bolstered by sub-clonal variations within these clonal populations [2], much like the heterogeneity observed between individuals of a species in nature. Advances in single cell analysis have provided an unprecedented look into the clonal and sub-clonal architecture of cancer [3] and uncovered considerable intra-tumor heterogeneity (ITH) at multiple biological levels. For example, tumors often show extensive cell-to-cell heterogeneity in epigenetic markers, gene mutations, and chromosome aberrations, as well as spatial heterogeneity in the conditions of the extracellular microenvironment [4,5,6]. Heterogeneity in one or more of these components can be associated with poor patient outcomes [4,5,6,7,8] and increased probability of disease recurrence [9,10,11,12,13]. Not surprisingly, these forms of heterogeneity underlie marked cell-to-cell heterogeneity in a range of phenotypes, including differences in protein biomarker expression, proliferation, cell and nuclear morphology, immune cell infiltration, motility, metabolism, angiogenic potential, differentiation status, and metastatic potential [14,15,16,17].

Cell-to-cell heterogeneity emerges through evolutionary processes, in which new variants are generated by ongoing molecular changes and either survive or are eliminated by natural selection. Epigenetic changes are common in cancer and can occur in response to changes in the extracellular environment or due to perturbations in the cellular machinery that orchestrates epigenetic regulation [18]. For example, mutations or altered expression of genes involved in epigenetic regulation (e.g., regulating DNA methylation, histone modifications, and regulatory non-coding RNAs) can lead to increased rates of epigenetic change (known as epigenetic instability) and epigenetic heterogeneity in tumors [19,20,21,22,23,24,25]. Increased rates of mutation at the DNA sequence or chromosomal level, a phenomenon collectively known as genomic instability (GIN), occurs in the vast majority of tumors [26]. The rate of gene mutation can increase due to defective DNA damage repair (mismatch repair, nucleotide excision repair, homologous recombination), DNA replication stress, or structural damage to the chromosomes [8]. Chromosomal abnormalities are also widely observed in tumors [27]. These aberrations emerge through defective chromosome segregation or chromosomal damage (leading to gain or loss of whole or partial chromosomes, known as aneuploidy), or abnormal cell cycle events that lead to genome doubling (polyploidy) [28,29]. Chromosomal instability (CIN) refers to the form of GIN where numerical and/or structural chromosomal aberrations occur at an increased rate. 

CIN has been reported as being the most common form of genomic instability in human cancers [30,31,32], and both CIN and aneuploidy are present in most human tumors [27,33,34,35]. Despite the complexity involved with untangling the cellular effects of aneuploidy, studies in various model systems have made substantial progress in uncovering how chromosomal aberrations alter cell physiology. In addition to gene-specific effects associated with gain or loss of specific chromosomes or chromosome fragments, aneuploidy and polyploidy in general are associated with a number of cellular effects, including substantial alterations to proliferation rates, cellular metabolism, protein homeostasis, and other phenotypes (reviewed in [36]). Aneuploidy and polyploidy have each been shown to drive tumorigenesis in certain circumstances [37,38,39,40,41,42]. Large scale chromosome or genome level alterations, such as aneuploidy and polyploidy (hereafter referred to as karyotype aberrations), are expected to have a larger penetrance (i.e., more likely to have a phenotypic effect on the cell) than most sequence-level events [8]. Furthermore, chromosome copy number changes affect a larger portion of cancer genomes than any other form of mutation [43]. Therefore, this review will examine the role of karyotypic heterogeneity (i.e., chromosome copy number heterogeneity) in cancer, as well as the environmental context surrounding karyotype aberrations (for excellent reviews addressing sequence-level and epigenetic heterogeneity, please see [8,18,44,45]). 

There is a growing appreciation for the context-dependent (genetic, physiological, environmental, etc.) effects of karyotype aberrations on cell physiology and in cancer (reviewed in [46]). Aneuploid and polyploid cells can cause changes in the cellular and tissue environment [47,48,49], which may disrupt the normal contextual cues from the local environment that maintain tissue homeostasis. The maintenance of tissue homeostasis serves as a barrier to tumorigenesis [50,51], and deteriorating tissue health may create opportunities for cancer development. Although the importance of genomic and environmental changes in cancer development are generally accepted [7,52], our understanding of the details and ramifications of the interplay between genomic and environmental alterations is far from complete. The goal of this review is to discuss the causes and consequences of karyotype aberrations from the perspective of both the cell and the extracellular environment. We will focus on the role of aneuploidy and polyploidy within the context of tumorigenesis, specifically addressing factors that lead to the accumulation of aneuploidy, the effects of karyotype changes on intercellular and environmental interactions, and the disastrous impact this may have on the tumor microenvironment (TME) and cancer evolution.

## 2. Cellular Routes to Karyotype Change

**Figure 1:**
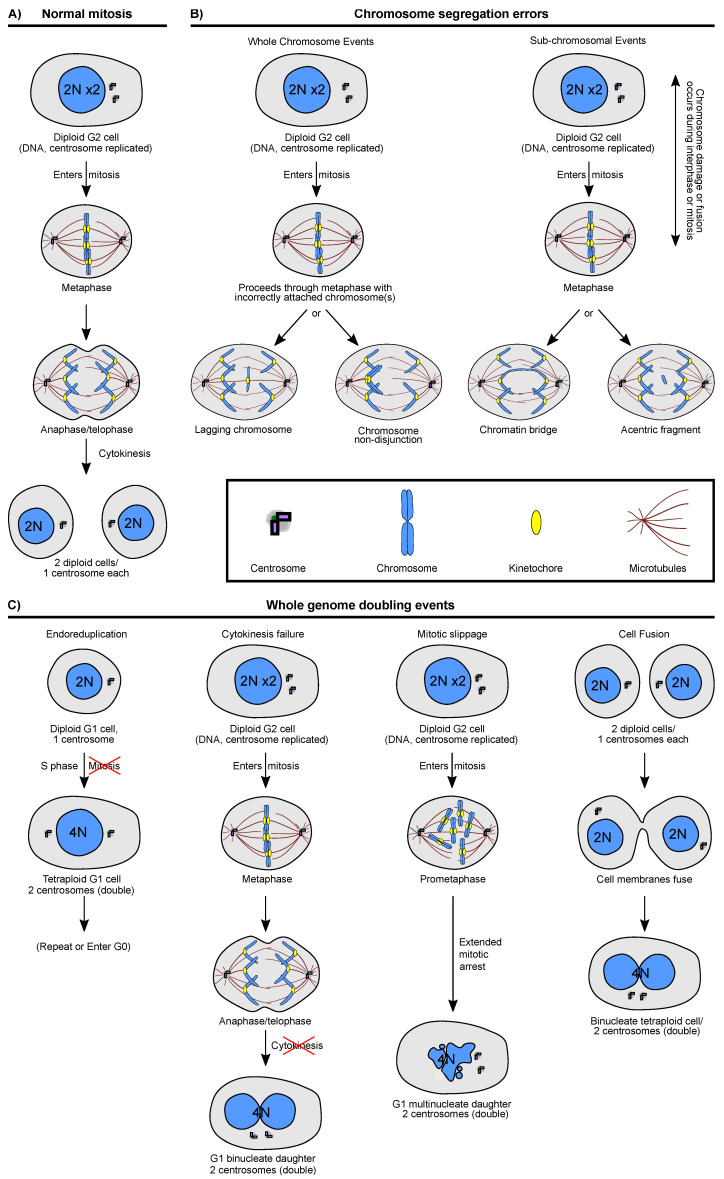
Cellular mechanisms leading to karyotype aberrations. Examples of (**A**) a normal mitosis and (**B**) abnormal mitoses leading to the missegregation of whole chromosomes (lagging chromosomes and chromosome non-disjunction; left column), chromosome fragments (right column, right daughter cell), or chromatin bridge-mediated chromosome missegregation (chromatin bridge, right column, left daughter cell; which can give rise to a variety of outcomes, including aneuploidy and tetraploidy [79,84]). Lagging chromosomes, chromatin bridges, and acentric fragments can all give rise to cells with micronuclei. (**C**) Examples of whole-genome duplication events, including endoreduplication, cytokinesis failure, mitotic slippage, and cell fusion (left to right).

Several mechanisms that can lead to karyotype changes have been well described (reviewed in [28,29]), and include events that can lead to gains and losses of individual chromosomes as well as events that lead to doubling of the genome (Figure 1). Whole genome duplication (WGD) events can occur by a number of different mechanisms, including cell fusion (two cells of the same or different type fuse), cytokinesis failure (a cell proceeds through mitosis, but fails to complete cytokinesis), mitotic slippage (a cell aberrantly exits mitosis without chromosome segregation), and endoreduplication (a cell proceeds through successive S-phases without intervening mitoses) (Figure 1C) [53,54]. The specific route of genome doubling may have different consequences for the cell. For example, mitotic slippage leads to nuclear envelope defects and DNA damage while the other mechanisms are less likely to do so [55]. Newly formed tetraploid cells also inherit extra centrosomes, which can disrupt spindle formation (e.g., leading to multipolar divisions) and kinetochore-microtubule attachments in subsequent divisions [56,57].

Whole chromosome gains or losses generally arise through missegregation of chromosomes in mitosis, leading to an unbalanced inheritance of genomic information by the two daughter cells (Figure 1B, left column). Whole chromosome missegregation can occur via multipolar divisions. While multipolar divisions in tetraploid cells lead to highly aneuploid karyotypes with chromosome counts in between diploid and tetraploid—as observed in tumors [58]—they also lead to a very high likelihood of losing most or all copies of at least one chromosome [59] and daughters of multipolar divisions are rarely viable in cell culture [57,59]. Whole-chromosome missegregation can also occur due to erroneous attachment of the sister chromatids (via the kinetochore) to the microtubules of the mitotic spindle. Such errors include chromosome non-disjunction and anaphase lagging chromosomes. Chromosome non-disjunction occurs when both sister chromatids are segregated into one daughter cell when their kinetochores are both attached predominantly (mero-syntelic attachment) or solely (syntelic attachment) to microtubules from one spindle pole [60,61,62,63]. Chromosome non-disjunction may also occur if spindle assembly checkpoint function is compromised and cells enter anaphase with monotelic attachments (one sister kinetochore is attached to a spindle pole while the other kinetochore is unattached) [64,65]. Anaphase lagging chromosomes are another example of chromosome missegregation and they occur when a single kinetochore is attached to microtubules from two spindle poles (merotelic attachment), causing the chromosome to lag behind the other chromosomes in anaphase. Lagging chromosomes may segregate into either daughter cell and rejoin the main chromosome mass before nuclear envelope reformation, resulting in either aneuploidy or euploidy [60,66]. However, lagging chromosomes often lead to the formation of micronuclei, where the nuclear envelope reforms separately around the main chromosome mass and the lagging chromosome(s).

Chromosomes in micronuclei undergo DNA damage at higher rates than chromosomes in the main nucleus [67,68], in part because of defective nuclear envelopes in micronuclei [67,69,70] and erroneous mitotic DNA replication [71]. Chromosomes in micronuclei have been observed to undergo large scale damage (shattering), leading to complex structural re-arrangements of chromosomes in a short time period, a process known as chromothripsis [68,72,73]. Furthermore, a study in PtK1 cells found that chromosomes from micronuclei were more likely than those in the main nucleus to missegregate in the following cell division [74]. Thus, lagging chromosomes can result in no karyotype change, or can lead to whole chromosome aneuploidy, structural aneuploidy, or both. Aneuploidy can also arise due to DNA damage, often accompanied by aberrant DNA repair (Figure 1B, right column). DNA damage can break chromosomes, which can lead to missegregation of chromosome fragments, unbalanced chromosomal translocations, and other partial chromosome copy number changes. Chromatin bridges—a result of chromosome fusion after DNA breaks, telomere dysfunction, or failure to completely replicate or decatenate DNA—often result in structural karyotype aberrations [71,75,76,77,78,79], but can also lead to polyploidy and whole chromosome aneuploidy [79,80,81]. Along with lagging chromosomes, chromatin bridges and acentric fragments can give rise to cells with micronuclei, which mark the occurrence of chromosome segregation errors [82,83].

## 3. Environmental Causes of Karyotype Change

The mechanisms leading to karyotype change discussed above can arise due to spontaneous cellular errors. However, conditions in the extracellular environment can increase the frequency of aberrant mitoses. Various environmental stresses can induce gene mutations or CIN [85,86,87,88,89,90,91]. The specific effects of these stresses are modulated by the nature, magnitude, and duration of the stress. Both endogenous (physiological) and exogenous stressors may contribute to genome instability in this way.

Chronic inflammation, which can result from hereditary conditions, diet, and environmental exposure to toxic substances or infectious agents, is a major risk factor for cancer development [92]. Many precancerous lesions (such as Barrett’s esophagus, inflammatory bronchial lesions, and ulcerative colitis) are closely associated with both inflammation and karyotype aberrations [93,94,95]. Notably, inflammatory factors have been causatively linked to aneuploidy [96,97,98] and micronucleus formation [99] in some systems. Several mechanistic links between inflammation, DNA damage, and chromosomal aberrations have been reported. One study found that misexpression of activation induced cytidine deaminase (AID), induced by inflammation-mediated NF-κB signaling, can lead to DNA double strand breaks, somatic mutations, and chromosomal aberrations [100]. Several matrix metalloproteases (MMPs) are also increased in inflamed tissue [101], and expression of MMP-3 and MT1-MMP have been linked to increased CIN [102,103,104]. Furthermore, inflammation can induce epithelial-to-mesenchymal transition (EMT) in cancer cells both by direct action of soluble mediators of cancer-associated inflammation (TGF-β, TNF-α, IL-1β, IL-6, IL-8, CCL2, among others) and by the action of various types of immune cells including M2-activated tumor associated macrophages (TAMs) [105]. It has been shown that cells undergoing EMT can fail cytokinesis and become chromosomally unstable if they fail to arrest [106]. Finally, inflammation can promote genome instability by inducing oxidative stress [101]. Oxidative stress—which may be the product of inflammation or factors such as metabolic dysfunction or radiation [107]—causes damage to various cellular components, including DNA. Oxidative stress is associated with oxidation of DNA bases, induction of DNA double strand breaks, gene mutation, and structural aberrations of the chromosomes [108,109,110,111,112]. Chronic oxidative stress has also been reported to lead to loss of telomere function and, possibly as a consequence, polyploidization [112,113]. Therefore, chronic inflammation and/or oxidative stress can have mutagenic, clastogenic, and aneugenic effects on cells that reside within the inflamed tissue or tumor.

Other extracellular conditions have also been observed to promote genetic or chromosomal changes in cultured cells, including serum starvation, hypoxia, lactic acidosis, irradiation, and exposure to DNA damaging agents [86,87,88,114,115,116,117]. These factors represent stresses that can occur in tissues or tumors under certain conditions but are largely atypical in healthy tissues. A variety of exogeneous biotic and abiotic factors have also been linked with karyotypic changes, including viral infection [118,119,120] and exposure to chemicals [121,122]. Viruses or mutagenic agents can also lead to gene mutations or gene inactivation, which may be permissive for the proliferation of aneuploid or polyploid cells (such as inactivation of p53) [123,124]. A number of other studies have linked chemical exposure (e.g., bisphenol A (BPA), heavy metals, air pollution) with accelerated telomere attrition [125,126,127], which promotes chromosome fusions and breakages, polyploidization, and aneuploidy [128,129].

Tissue architecture is critically important for the maintenance of euploidy. Loss of tissue architecture was shown to lead to mitotic errors and aneuploidy in mouse epithelial cells [130]. Total loss of substrate adhesion was also found to promote cytokinesis failure [131], and wound healing is also associated with emergence of tetraploid cells [132]. Interestingly, loss of substrate adhesion was also found to reduce p53 expression [133,134], which may enable the survival of both aneuploid and polyploid cells [135,136]. Aging—one of the most potent risk factors associated with cancer—is associated with deteriorating tissue architecture [137,138], suggesting that one link between aging and cancer could be a loss of some karyotype-protective features found in younger tissues. Indeed, aneuploid and polyploid cells in the body have been reported to accumulate with age [139,140,141,142], although this claim has been disputed [143]. Altogether, these studies show that the body and tissue environment are critical factors in preventing the genesis of abnormal cells and that a number of factors—including aging, chemical exposure, inflammation, and exposure to harmful chemicals or biological agents—can destabilize cellular mechanisms for maintaining genome integrity.

## 4. Aneuploidy and Polyploidy Can Both Promote and Buffer Karyotypic Heterogeneity

Aneuploidy, karyotypic heterogeneity, and CIN correlate with several parameters of disease progression, including drug resistance [9,144,145,146,147,148,149], metastasis risk [150,151,152,153,154,155,156,157], and clinical outcome [6,158,159,160,161,162,163]. While in many cancers the degree of CIN correlates with degree of aneuploidy and karyotypic heterogeneity [164,165,166,167], the relationships between CIN, aneuploidy, and heterogeneity can be complicated. Highly aneuploid tumors are sometimes observed in the absence of ongoing CIN and, conversely, tumors displaying CIN are not always highly aneuploid or karyotypically heterogeneous [30]. The rate at which new karyotype aberrations arise is certainly an important piece of the equation for determining the extent of karyotype heterogeneity in a tumor, but it is balanced by the ability of cells to tolerate new karyotypic aberrations and selective pressures from the environment. Therefore, the amount of karyotypic heterogeneity in a population is a function of the rates at which cells with novel karyotypes are generated and eliminated.

**Figure 2:**
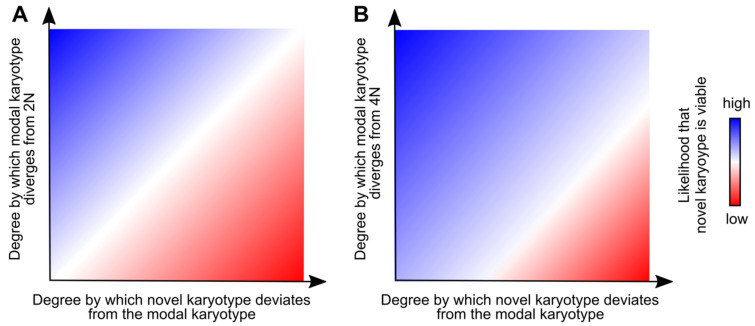
Aneuploidy and polyploidy increase the ability of cells to tolerate mitotic errors and resulting karyotype aberrations. As populations of diploid cells (**A**, origin) evolve to become more aneuploid (move up the y-axis), the degree by which novel karyotypes can diverge from the modal karyotype and result in viable cells increases (“permissive zone”, represented roughly by the size of the blue zone at the given height). This would be expected to increase the amount of karyotypic heterogeneity in a cancer cell population and, in turn, its evolutionary potential. (**B**) Tetraploidy buffers against negative fitness effects caused by aneuploidy. Therefore, near-4N cells are expected to have a larger permissive zone than their near-2N counterparts, which may explain why whole genome doubling increases karyotypic heterogeneity and is a favorable route to complex aneuploid karyotypes.

For karyotypic heterogeneity to accumulate, cells must tolerate either ongoing or punctuated bursts of mitotic errors. But what determines if a cell will survive and contribute to karyotypic heterogeneity? The type of error that occurs can affect the cellular outcome (Section 2). The ploidy of the cell in which karyotypic aberrations occur is also important for determining their effects. Aneuploidy already established in a mother cell appears to be associated with reduced fitness cost of additional chromosome missegregation (i.e., aneuploidy tolerance) and with more karyotypic variation [168]. In organoids derived from colorectal cancers, the degree of aneuploidy was, indeed, found to correlate with the ability of cells to tolerate mitotic errors and with karyotypic heterogeneity [164]. In a study of paired primary and metastatic cancer cell lines, the amount of karyotypic variation from cell to cell (i.e., “karyotypic divergence”) was higher in the more aneuploid cancer cells [169]. For example, a near-diploid breast cancer trisomic for chromosomes 7 and 10 (modal chromosome number of 48) had one non-clonal chromosome aberration per cell on average with a range from 0–5, whereas a highly aneuploid pancreatic cancer (modal chromosome number of 64) averaged 10 non-clonal chromosome aberrations per cell with a range from 0-26 [169]. Consistently, an analysis of the Mitelman database found that near-triploid tumors displayed more intercellular karyotype variability compared to near-diploid tumors [170]. Similar to aneuploid cells, tetraploid cells are more tolerant of mitotic errors and accumulate more karyotypic heterogeneity than their diploid counterparts in cell culture [171]. Tetraploidy may offset the high fitness cost of chromosome gains and losses by doubling the copy number of each chromosome [172,173,174]. This aneuploidy tolerance may explain why WGD often occurs in the evolution of tumors with complex karyotypes. In line with this theory, Dewhurst et al. reported that a majority of colorectal cancers with near-triploid karyotypes evolved through a tetraploid intermediate and displayed more genomic complexity than near-diploid tumors [175]. Together, these studies suggest that polyploidy and/or the degree of aneuploidy may increase the margins of viable karyotype variation, or the “permissive zone” for which cancer cell karyotypes can diverge from the modal karyotype and survive (Figure 2).

Another important factor for karyotypic heterogeneity is the rate of chromosome segregation errors. While aneuploidy can provide a fitness advantage under some circumstances [176,177,178], aneuploidy may also lead to decreased fitness under normal growth conditions [178,179,180]. High levels of CIN can also lead to decreased cellular fitness and increased cell death, due to the emergence of cells with new and inviable karyotypes [181,182]. A mathematical model predicted that cancer cells will find an optimal chromosome missegregation rate, at which fitness costs due to missegregation and random, possibly detrimental, aneuploidies are balanced by the generation of phenotypic heterogeneity [173,183]. According to this model, if the rate of chromosome missegregation is too high, cell population growth becomes hampered by the frequent birth of daughter cells with inviable karyotypes. Conversely, not having enough CIN results in less karyotypic (and presumably phenotypic) heterogeneity, which reduces the tumor’s evolutionary potential. This model is supported by observations in mice and human tumors. Several clinical studies reported an association between high CIN and poor patient outcomes in several solid tumor types by categorizing patient tumors as either high or low CIN [161,162,184,185]. However, studies using a non-binary classification of CIN in breast tumors found that the highest levels of CIN were associated with improved patient outcomes [186,187]. Similarly, a parabolic relationship between CIN and patient outcome was observed in breast, ovarian, gastric and non-small cell lung cancers, such that tumors with intermediate levels of CIN had the worst prognosis and both low and high levels of CIN corresponded with better patient outcomes [188] (for further discussion on the relationship between CIN and clinical outcome, see [189]). In a mouse model, low-to-moderate levels of CIN were found to promote tumorigenesis, while high levels of CIN suppressed tumor progression [163]. As a result of these observations, it has been proposed that exacerbating CIN beyond a tolerable level may be a viable therapeutic strategy [190], but such an approach should be considered with caution [191,192]. 

Altogether, these findings suggest that the coupling of an optimal CIN rate with sufficient aneuploidy to tolerate ongoing karyotypic variation appears to create ideal conditions for cancer evolution.

## 5. The Role of Aneuploidy and Polyploidy in Tumor Niche Construction

**Figure 3:**
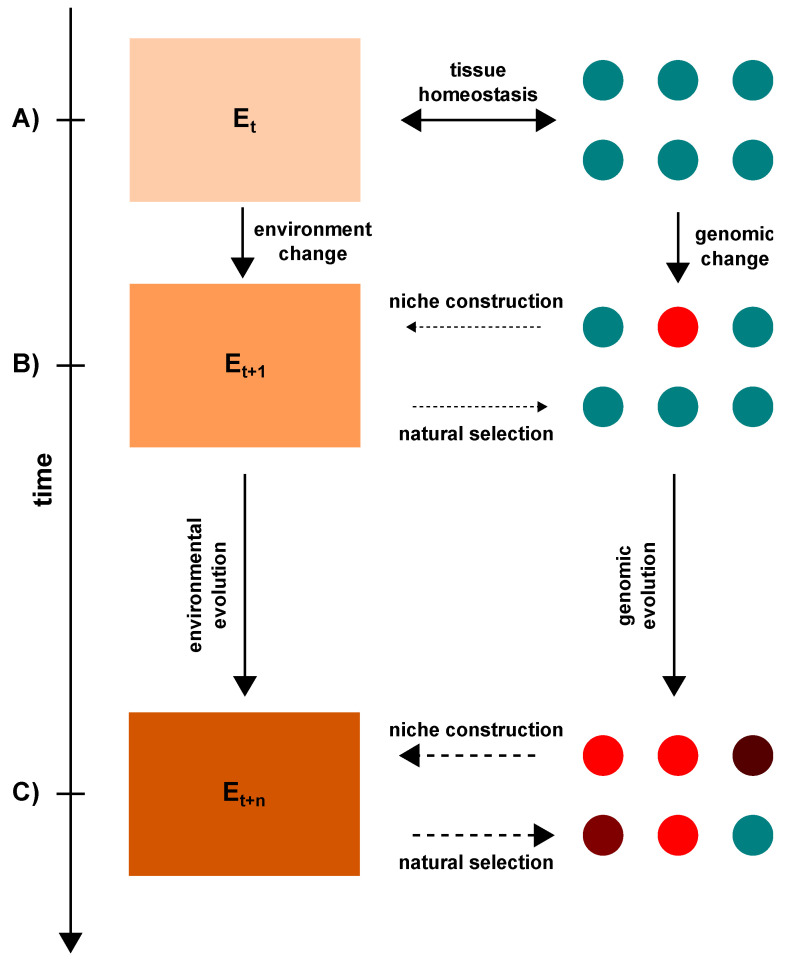
Bidirectional, cell-environment interplay in tumor niche construction and the genomic evolution of cancer cells. (**A**) In normal tissues, cells and the environment interact to promote homeostasis by regulating cell growth, division, and other behaviors essential for proper health. Teal circles depict normal diploid cells and beige-colored square indicates a normal, healthy environment. (**B**) Over time, however, changes—either natural (aging) or from stress (smoking, obesity, inflammation, etc.)—may occur in either the cell or environment that disrupt this homeostasis. Spontaneous cellular errors may lead to genomic changes (red circle) that alter cell physiology and interactions with the environment, through senescence, cell death, or increased production of lactate, reactive oxygen species, and other signaling molecules, initiating the process of niche construction (thin dashed arrow). Alternatively, environmental conditions may change (light orange-colored square) that increase the frequency of mutations and mitotic errors in cells and select for cells with favorable genomic alterations and/or phenotypes (thin dashed arrow). The order of events that begin tumor niche construction can vary, starting from either a cellular or environmental change. (**C**) As this bidirectional interplay persists, genomic and environmental evolution continue to influence and shape each other. As the environment erodes and is replaced by a pro-tumorigenic one (dark orange-colored square), various stresses (hypoxia, acidosis, nutrient scarcity, etc.) may emerge that exert strong selective forces (thick dashed arrow) and favor the survival of tumor cells with advantageous genomic changes. In turn, the outgrowth of these abnormal cells amplifies their environmental effects (thick dashed arrow), which continue to modify selective pressures for their benefit. This cycle may serve as a destabilizing feedback loop that explains the substantial genomic and environmental alterations and heterogeneity (different colored circles) observed in malignant aneuploid tumors.

For a complete picture of the role of genomic changes in tumor progression, it is important to examine the bidirectional interplay between cancer cells and their environment, in which cells and tissue both determine and modulate the health of the other. This interplay unfolds throughout the evolutionary history of the tumor, molding and shaping both the TME and tumor cells into entities that are distinctly different than those found in normal tissues (Figure 3). This mirrors ecology’s “niche construction concept,” which describes the formation of ecological niches through the continuous interplay between selection of individuals by the environment and the modification of the environment by the individuals [193,194,195]. Mathematical modeling and experimental observations of natural systems in which niche construction is an acting force demonstrate that it can alter the evolutionary trajectory of populations [194,195,196,197] and the spatial patterning of individuals in an environment [195,197,198,199]. In tumors, niche construction by cancer cells often results in harsh environments, such as areas with low pH (acidosis) and/or oxygen (hypoxia), that may favor the growth of malignant cells over non-malignant cells. As we have discussed, genomic changes may result from perturbations in the environment (Section 3). There is also evidence that aneuploid and polyploid cells actively remodel their local environment and may have an advantage compared to diploid counterparts in stressful conditions [171,178]. These findings along with the widespread nature of aneuploidy and abnormal environmental conditions observed in human tumors hint at a relationship between aneuploidy and tumor niche construction, although much remains to be learned about this possible link. In this section, we will explore this subject further by examining the role of aneuploid and polyploid cells in shaping the TME (Section 5.1) and the role of the TME as a selective force on karyotypically heterogeneous cells in tumors (Section 5.2).

### 5.1. Environment Remodeling by Aneuploid and Polyploid Cells: Home Is Where You Make It

While changes in the local environment may cause cell stress and genomic alterations, cells can also shape their own environmental niche through complex interactions with other cells, the extracellular matrix (ECM), and the secretion of signaling molecules or metabolites [200,201]. Cancer cells often harbor a myriad of gene mutations, epigenetic modifications, and karyotypic abnormalities that drive tumorigenesis [26], making it difficult to attribute any environmental effects to a specific oncogenic event. To avoid such confounding factors, much of our understanding about the cellular consequences of aneuploidy comes from carefully controlled experiments that use yeast and mammalian cells with single (or few) aneuploidies or induce short pulses of chromosome missegregation by perturbing the mitotic checkpoint. Aneuploid and polyploid cells have been found to exhibit a diverse spectrum of biological changes, including altered cell fitness, metabolism, and gene expression (reviewed in [36,202,203,204]). While some of the physiological effects associated with aneuploidy may be specific to the loss or gain of a certain chromosome and not others, a number of studies have found that some physiological effects of aneuploidy are independent of the identity of the particular chromosome being gained or lost. These studies have provided various lines of direct and indirect evidence suggesting that the physiological changes brought about by CIN, aneuploidy, or polyploidy are important in shaping the cell’s relationship with its surroundings. Here, we discuss how the known cellular effects of karyotype aberrations, while only one of the important players in tumor formation, may have potent effects on the environment that disrupt tissue homeostasis and contribute to the co-evolution of cancer cells and the TME observed throughout disease progression [50,205].

#### 5.1.1. The Transmission of ER Stress to Immune Cells Impairs Anti-Tumor Immunity

Aneuploidy has been found to elicit characteristic cellular stress responses regardless of which chromosome is affected. For example, stoichiometric mismatches between subunits of protein complexes that are encoded on different chromosomes can lead to endoplasmic reticulum stress (ER stress) in aneuploid cells [206,207,208], and this appears to happen regardless of the specific chromosome that is gained or lost in human and yeast cells [206,208]. Cells experiencing ER stress release soluble molecules into the extracellular environment. These cell secretions can, in turn, induce an ER stress response in adjacent stromal cells and alter their behavior [209,210,211]. In one study, inducing ER stress in cancer cells elicited an ER stress response in macrophages in co-culture, which led to enhanced production of proinflammatory cytokines by the macrophages [210]. Similarly, the transmission of ER stress from cancer cells to dendritic cells led to arginase activation and impaired T cell function [211]. In mice, ER stress in dendritic cells resulted in constitutive XBP1 activation and altered lipid homeostasis, which repressed T cell-dependent anti-tumor immunity and promoted ovarian cancer progression [209]. An analysis of chromosomal alterations in TCGA samples across 32 tumor types found that aneuploidy positively correlated with gene expression associated with ER stress and the unfolded protein response (UPR), but negatively correlated with intra-tumor T cell cytolytic activity [212]. Furthermore, the same study found that inducing aneuploidy in pseudodiploid cancer cell lines and polyploidy (via cell fusion) in mouse embryonic fibroblasts (MEFs) triggered ER stress. Strikingly, exposure of macrophages to conditioned media from these aneuploid cells promoted an immune-suppressive and proinflammatory phenotype [212]. Altogether, these findings suggest that aneuploidy-induced ER stress may play an important role in repurposing the TME to fuel cancer progression, particularly through altering the function and behavior of immune cells in the tumor microenvironment.

#### 5.1.2. Changes in Metabolism and ROS Homeostasis May Contribute to Tumor Acidosis and Inflammation

Metabolic alterations are commonly observed in aneuploid and polyploid cells [180,213,214,215,216,217,218]. Both aneuploidy and polyploidy lead to increased glycolytic activity and lactate production [215,217,218,219,220]. Metabolic byproducts, such as lactate, are thought to be a major contributor to tumor acidification [221]. Therefore, it is plausible that increased lactate production by cells with abnormal karyotypes could promote the acidification of the extracellular environment during tumor formation, but this link has not been experimentally validated in vivo. Acidosis is common in tumors and can have profound effects on the ongoing cell-cell and cell-environment interactions in the TME. Low extracellular pH disrupts immune system interactions with cancer cells, promotes tissue remodeling, invasion, and metastasis [221,222,223,224]. Aneuploid yeast, human, and rodent cells in vitro have been reported to harbor numerous other metabolic changes, including increased glutamine uptake, increased production of ammonium and glutamate and altered nucleotide and sphingolipid metabolism [215,216,219,220]. The metabolic composition of tumor interstitial fluid was recently characterized for several murine tumor types and compared to levels in circulating plasma. The composition of the two fluids was found to differ considerably, due to the rates of nutrient influx via circulation, consumption of nutrients and excretion of metabolic byproducts by cells, and the clearing of metabolic waste into circulation [225]. It is not clear how the altered metabolism of aneuploid or polyploid cells may influence the composition of the interstitial fluid, or what functional consequences this may have for tumor evolution. However, given the observations of altered metabolism in cells with karyotype aberrations, this may be an interesting and important question to answer.

Aneuploid and chromosomally unstable cells show increased levels of reactive oxygen species (ROS) [214]. Multiple mechanisms may contribute to the elevated ROS production in aneuploid cells. Ca^2+^ release from the endoplasmic reticulum, which occurs following prolonged activation of the UPR during ER stress, can interfere with the electron transport chain, lower mitochondrial integrity, and increase ROS levels [226,227]. Furthermore, increases in the number and activity of mitochondria in cells after the induction of CIN may also lead to the accumulation of ROS [214,228,229]. Higher ROS levels are common in the TME and can promote oxidative stress in cancer and stromal cells [230,231]. While oxidative stress is associated with genotoxicity, protein damage, and mitotic errors [232,233,234], it also affects how cells interact with their surroundings. In cancer-associated fibroblasts (CAFs), for example, oxidative stress leads to excessive production of lactate, ROS, and nitric oxide, which can increase aneuploidy in adjacent cancer cells [235]. Oxidative stress can also induce inflammation, another driver of cancer development, which can cause DNA damage and CIN [101,236]. Inflammation, in turn, can trigger recruitment of leukocytes, such as neutrophils, lymphocytes, dendritic cells, and macrophages [237]. Although an immune response can eliminate cancer cells, these immune cells can also secrete potent growth factors that promote angiogenesis and potentiate cancer progression [238].

#### 5.1.3. CIN, Cell Death, and Senescence: Potent Forces in Tissue Niche Construction

CIN can lead to the birth of cells with reduced fitness and an increase in cell death owing to the inheritance of complex, and sometimes inviable, karyotypes with random aneuploidies. Cell death has been found to cause the release of stimulatory factors that promote the proliferation of nearby cells [239,240], as well as inflammation and immune cell recruitment [241,242]. Indeed, increased proliferation along with increased cell death (i.e., high cell turnover rate) in tumors may signal a more aggressive disease [243,244]. Complex karyotypes and/or micronuclei formation resulting from CIN can also cause cell cycle defects, DNA damage, and/or stress-induced cell senescence [181,182,245]. The latter is especially important to consider, as senescent cells can have powerful effects on the local environment. Senescent cells exhibit a secretory phenotype (known as the ‘senescence associated secretory phenotype’, or SASP), which can be associated with tumor progression [246,247]. Secreted SASP proteins, which include growth-promoting factors, cytokines, and chemokines, have been shown to promote cell proliferation, inflammation, cell differentiation or phenotype switching, tissue remodeling, angiogenesis, and invasion [246,247]. Senescent cells can also help neighboring cells escape immune detection by cleaving cell surface receptors both in Natural Killer (NK) cells and their potential target cells [248,249]. The enrichment of senescent cells at the invasive front compared to the tumor center in breast [181] and papillary thyroid carcinomas [250] suggests that SASP-mediated environmental remodeling may be important for tumor invasion. Furthermore, increased levels of tetraploidy and karyotypic heterogeneity have also been observed at the tumor margins relative to the core [181,251]. Why tumor cell senescence, WGD events, and CIN may occur more frequently at the tumor margins is unclear but could stem from the environmental conditions (and/or the need for environmental remodeling) and interactions between cancer and stromal cells in these regions.

Micronucleus formation due to chromosome missegregation can also trigger inflammatory signaling [156,252,253]. When micronuclei containing missegregated chromosomes rupture, genomic DNA is exposed to the cytoplasm and activates the cGAS-STING pathway, which can lead to non-canonical NF-κB signaling, EMT, and metastasis [156]. This same study also found that cancer cells with a high rate of CIN displayed mesenchymal cell traits, including increased motility, invasiveness, and vimentin expression [156]. Changes in the levels or spatial organization of vimentin, an intermediate filament involved in cell adhesion, in cancer cells can lead to the stiffening of tumor tissues and alter the biomechanical properties of the TME [254,255,256]. Reducing CIN levels or micronuclear rupture delayed metastasis in aneuploid tumors [156], demonstrating that the environmental effects associated with cGAS-STING activation, chronic inflammation, and altered tissue stiffness—rather than the karyotypic alterations alone—are important for cancer progression in this system. Importantly, these changes are independent of aneuploidy, indicating that lagging chromosomes can contribute to cancer progression and niche construction via micronucleus formation even if the lagging chromosome is ultimately segregated into the correct daughter cell. CIN and micronucleus formation, however, do not always cause EMT or promote invasive behavior, even if cGAS-STING is active [257]. Similarly, micronucleus formation does not always lead to cGAS activation [258]. One study found that chromatin bridges, but not micronuclei originating from whole chromosomes, activated cGAS, resulting in the spread of inflammatory signaling from cancer cells to stromal cells (fibroblasts and monocytes) in a co-culture model [258]. Therefore, while the effects of CIN and micronucleus formation on EMT and cGAS-STING activation appear context-dependent, both whole chromosome missegregation and chromatin bridges may induce a chronic inflammatory response that fuels tumor progression.

#### 5.1.4. Altered Centrosome Homeostasis Affects Tissue Organization, Invasiveness, and the Cellular Secretome

Karyotypic aberrations have also been associated with altered centrosome homeostasis [54,172,259,260] and, while causation has not been demonstrated experimentally, it has been proposed that aneuploidy may lead to disrupted centrosome homeostasis [261]. Importantly, similar to aneuploidy, extra centrosomes and structural centrosome abnormalities are common features in human malignancies [262]. It has been shown that, in some contexts, extra centrosomes by themselves are sufficient to promote tumorigenesis [263,264]. Extra centrosomes may contribute to cancer progression by promoting CIN and therefore more aneuploidy [56,57]. Besides promoting CIN, extra or abnormal centrosomes can promote behaviors that alter their microenvironment directly. Experimentally induced centrosome structural defects, meant to mimic changes seen in cancer cells, disrupted tissue organization in 3D cultures [265] and increased invasiveness [266]. Furthermore, extra centrosomes have been linked to a secretory phenotype very similar to that observed in senescent cells which increased invasiveness in nearby cells [267]. Finally, tumors derived from the injection of tetraploid cells into mice had high levels of centrosome amplification and high expression of MMPs [37], which modify the ECM and the extracellular surfaces of other cells and increase cellular invasiveness. The full nature of the link between polyploidy and centrosome amplification remains unknown, however, as polyploid cells in culture quickly lose extra centrosomes [57,84,171,266,268].

#### 5.1.5. Aneuploid Stromal Cells May Also Alter the Tumor Microenvironment

Within a tumor, karyotype aberrations are not exclusive to the cancer cells and have been detected in a variety of cell types in the tumor stroma. While it is recognized that cancer-associated stromal cells have distinct phenotypes compared to their normal counterparts, the effects of aneuploidy on stromal cell behavior and their interactions with the TME is less clear. Chromosomal abnormalities and centrosome amplification have been reported in tumor-associated endothelial cells (TECs) as a result of hypoxia-induced oxidative stress, increased ROS production, and excessive pro-angiogenic signaling in the TME [269,270,271]. Interestingly, aneuploid TECs were morphologically distinct from normal endothelial cells, including differences in nuclear and cell size [270], which could contribute to the structurally abnormal and leaky blood vessels seen in tumors [272,273]. Defective vasculature, leading to inconsistent nutrient delivery and waste removal, is a major cause of hypoxic and acidic environments in tumors. Nevertheless, it remains uncertain to what extent aneuploidy in TECs contributes to these abnormal phenotypes. Some studies reported that CAFs, one of the most abundant stromal cell populations in solid tumors, are diploid and do not acquire genetic changes [274,275], while other studies have reported chromosome and gene copy number alterations in CAFs derived from melanoma, breast, prostate, colorectal, and ovarian cancer [276,277,278,279,280]. Nevertheless, loss of heterozygosity (LOH) due to changes in chromosome copy number or focal deletions in breast cancer CAFs at the genetic loci of *EP300*, *ATM*, *IL2RB*, and *IBD5*, which play a role in neovascularization, cell adhesion, ECM organization, and immune cell recognition, were associated with higher tumor grade and metastasis [280]. Together, these studies suggest that genomic alterations in TECs and CAFs may be an important feature of a tumor’s ecological landscape and contribute to disease progression.

#### 5.1.6. Environmental Remodeling by Aneuploid and Polyploid Cells—Summary

Together, the observations discussed here show that the diverse physiological effects of aneuploidy, polyploidy and CIN can lead these cells to alter the extracellular environment in various ways (Table 1). Aneuploidy- and/or polyploidy-associated changes in cell physiology include changes in stress response, metabolism, and centrosome homeostasis, each of which can manifest independent of the specific chromosome(s) gained or lost. Various lines of direct and indirect evidence suggest that these changes can contribute to tissue environment remodeling in ways that may influence tumor evolution. In light of these studies, we can theorize that optimal degrees of aneuploidy, CIN, and centrosome amplification may create a perfect storm for tumor evolution by allowing the evolving cell population to explore new karyotypes and phenotypes, and by producing a substantial level of inviable or senescent cells that release stimulatory and pro-tumorigenic factors into the local environment. In doing so, the emergence of more abnormal and aggressive cells may occur while the homeostatic mechanisms of normal tissues may simultaneously be eroded and replaced by a pro-tumorigenic, genome-destabilizing environment (Figure 3). Although we focused our discussion on the effects of aneuploidy in general, genetic, epigenetic, or chromosomal events that affect specific chromosomes, genes, or processes also have the potential to promote tissue remodeling. For instance, cells with oncogenic *KRAS* mutations have been observed to potently alter their surroundings and mediate cancer progression [281]. HCT-116 cells with trisomy 5 induced a partial EMT phenotype resulting in increased invasive and metastatic behavior, while gains of other chromosomes suppressed these phenotypes [257]. Moreover, specific chromosome arm copy number changes were associated with differences in leukocyte infiltration as well as macrophage polarity, although the cellular basis for these observations is unclear [282]. However, the value of karyotype aberrations in environmental remodeling may be especially relevant in tumor progression as these effects arise from general and common phenomena (aneuploidy, polyploidy, chromosome missegregation) and do not rely on specific aberrations, which may arise much less frequently. Because of the complex nature of cancer biology, it is important that these connections be interrogated with rigorous studies to better understand the role of aneuploid and polyploid cells in shaping the tumor niche(s) that drive tumor evolution.

### 5.2. Rigged Selection? Stress Conditions in the TME May Favor the Growth and Survival of Karyotypically Abnormal Cells

For niche construction to formally be said to occur, two conditions must be met: (1) an entity must engage in some activity to alter the environment and (2) the environmental change must modify the selective forces acting on that entity [197,288]. The TME, shaped by cancer cells throughout tumor evolution, does indeed exert selective pressures on cells that are very different than the forces that dictate cell survival in normal tissues [200]. In this section, we consider how selective pressures exerted by the constructed tumor microenvironment may favor the growth of karyotypically abnormal cancer cells. We also consider the role of stresses originating from outside of the evolving tumor—namely, cancer therapeutic treatments—in driving the actions of natural selection on aneuploid and/or polyploid cells.

#### 5.2.1. Karyotype Aberrations Can Confer Selective Advantage of Cancer Cells in Their Constructed Niches and in the Face of Cancer Therapeutics

Generally speaking, harsh or stressful environments (e.g., acidic, hypoxic, nutrient poor) eliminate cells that cannot tolerate them, allowing the proliferation and survival of those cells that are best adapted to the environment. Aneuploidy may provide a fitness advantage to various cell types under stress [9,289,290,291,292,293,294]. In some cases, specific aneuploidies may provide a selective advantage in a given environment by affecting the expression of important genes. The loss of chromosome 8p in MCF10A mammary epithelial cells promoted resistance to hypoxic conditions and chemotherapeutic drugs. This effect was attributed to increased autophagy linked to *ASAH1* LOH [295]. Although 8p loss was insufficient to induce transformation in MCF10A cells [295], it is commonly lost in human tumors of epithelial origin, which may be partly connected to the number of tumor-suppressor genes in that genomic region [296] as well as its effects on autophagy and lipid metabolism [295]. In human colon epithelial cells, trisomy 7 cells were found to out-compete diploid counterparts under serum starvation [176]. Similarly, the frequency of chromosome 7 copy number changes also increased in response to glucose deprivation and lactic acidosis in HCT-116 colorectal cancer (CRC) cells [297]. In a study using a different CRC cell line, DLD-1 cells harboring either an extra chromosome 7 or 13 showed more robust growth than euploid controls under conditions common in tumors, including hypoxia, nutrient starvation, and chemotherapy [178]. Notably, gain of 7p and 13q occur recurrently in CRCs [298], supporting the notion that these chromosomal changes may provide important contextual (genomic, transcriptional, environmental, etc.) advantages during colon carcinogenesis. In the case of trisomy 7, this karyotypic alteration may be favorable for cells in stressful environments due at least in part to dysregulation and/or amplification of the *EGFR* gene, which can maintain intracellular glucose levels and prevent autophagic cell death [299].

In many cases, the molecular mechanisms underlying the selective advantages of whole chromosome and chromosome arm aneuploidies are more complex (involving multiple genetic loci on different chromosomes) or unclear. For example, only 2 out of 64 chromosome arm alterations (CAAs) that were predictive of chemotherapeutic drug responses across cancer types could be explained by focal deletions of known drug targets [300]. This suggests that most CAAs associated with drug responses likely depend on the interaction of multiple genes across the affected genomic region and/or other interchromosomal genetic interactions. Following the induction of CIN, recurrent aneuploidies were observed in non-small cell lung cancer cells that developed resistance to the topoisomerase I inhibitor Topotecan [192]. The drug-resistant phenotype in this case was not driven by chromosomal alterations affecting the expression of the drug target. Instead, chromosome 6p gain caused the overexpression of resident genes *MAPK13* and *MAPK14* that encode for p38 kinase subunits, which led to the selective upregulation of a drug efflux pump on chromosome 4q [192]. Direct gain of 4q may not have been favorable in this context because it harbors numerous tumor suppressor genes, indicating that genetic interactions between specific aneuploidies and other chromosomes influence karyotype evolution (as reported in yeast [301]). In a similar study, recurrent aneuploidies were also detected in various cell lines following Mps1 disruption and drug pressure; however, the observed karyotypic changes were unique for each cell line used even when challenged with the same drug [191]. Although the mechanisms underlying resistance were not identified in this study, the unique karyotypic routes to drug resistance across cell types demonstrate there are multiple genomic paths to a given phenotype (drug resistance) and the cell’s genomic and/or epigenetic background is an important factor for the observed effects of chromosomal alterations.

There is also evidence that WGD can protect normal and cancer cells from stresses in the environment, including energy depletion, oxidative stress, and chemotherapy [171,302,303,304,305]. Polyploidy may be a major driver of treatment failure, tumor relapse, and drug-induced genomic evolution [306]. Multiple studies found that giant multi-nucleated polyploid cells arise in vitro and in vivo following drug exposure [307,308,309]. These polyploid cells may enter a reversible senescent-like state or slow cell cycle progression in response to drug treatment. While many of these cells may permanently arrest or perish [307], on some occasions, they undergo asymmetric, reductive divisions that produce mononuclear cells, which are often aneuploid and highly tumorigenic [310,311,312]. Furthermore, tetraploidy increased the resistance of non-transformed RPE-1 cells and HCT-116 CRC cells to a variety of chemotherapeutic drugs [171]. The effects of WGD may depend on the genetic background and/or mechanism of tetraploidization, as drug-induced mitotic slippage in PC9 lung cells did not promote resistance to the EGFR inhibitor gefitinib [191]. WGD can also render cells vulnerable to specific genetic challenges, such as impairment of DNA replication, proteasome inhibition, and KIF18A depletion [313]. Highly aneuploid cells (both WGD- and WGD+) were also more dependent on KIF18A compared to less aneuploid or euploid counterparts [314], indicating that KIF18A inhibitors may have immense therapeutic potential.

#### 5.2.2. Karyotypic, Genetic, and Epigenetic Changes Alter Selective Survival of Tumor Stromal Cells

Tumor stromal cells may also acquire important selective advantages through karyotypic changes. Karyotypic complexity and heterogeneity in TECs increased with tumor malignancy [315], and aneuploid TECs were more resistant to anti-angiogenic agents and chemotherapeutic drugs, such as vincristine, paclitaxel, and 5-fluorouracil, than normal endothelial cells [316,317]. Polyploid and aneuploid tumor-associated macrophages (TAMs) have also been detected in the blood of cancer patients [318,319]. By acquiring cancer cell DNA through phagocytosis, TAMs may gain tumorigenic functions that enhance tumor invasion and metastasis [318]. Recent studies found that CAFs isolated from premalignant and malignant skin squamous cell carcinoma were characterized by chromosomal abnormalities and genomic instability [320,321]. Katarkar *et al.* showed that CAFs with karyotype aberrations that amplified *NOTCH1* suppressed DNA damage-induced ATM signaling and cell cycle arrest in response to UV irradiation, promoting their survival over other CAFs [321]. Therefore, stromal cells with favorable genomic changes can indeed undergo positive selection during tumor progression, and the identification of such events could unlock new stroma-focused anti-cancer intervention strategies. This highlights the need for continued characterization of genetic, karyotypic, and epigenetic alterations in the tumor stroma and their effects on cancer-stromal cell interactions, which may underlie the clinical diversity in treatment response among tumors of the same class and stage [280].

#### 5.2.3. Karyotype Aberrations and Immune Interactions: A Matter of Context

The immune system’s role in eliminating damaged and abnormal cells represents an important selective pressure that cancer cells must overcome. The literature supports the idea that karyotype aberrations can modulate immune cell interactions, although the mechanisms and outcomes appear complicated and context dependent. Aneuploid cells in culture were found to be more susceptible than euploid cells to elimination by NK cells [322]. Similarly, it was shown that polyploid cells could be detected and eliminated by the immune systems of mice [323]. These findings suggest that the immune system may maintain tissue health and protect against cancer by detecting and eliminating aneuploid cells [324]. In humans, however, aneuploidy and polyploidy are associated with reduced immune cell infiltration in tumors, suggesting that aneuploidy may confer cells with a heightened ability to escape immune detection [35,313,325]. 

The mechanisms relating aneuploidy and immune interactions within tumors are not well understood, as highlighted by recent contrasting observations. One study found that aneuploid cells activated NF-κB signaling to promote their clearance by immune cells, and the NF-κB activity correlated with the degree of aneuploidy in cancer cell lines [326]. In clinical samples, however, highly aneuploid tumors had lower levels of NF-κB activity [35]. This discrepancy suggests that the suppression of NF-κB signaling may result from selective pressures imposed by the TME and represent an important event in the evolution of aneuploid cells in tumors. One explanation may lie in the link between aneuploidy, ER stress, and anti-tumor immunity (Section 5.1.1). ER stress, which is often induced by aneuploidy [206,207,208], has been associated with the down-regulation of MHC class I-associated peptides [327] and a reduced immune response in cell culture and mouse models [328]. Aneuploidy-induced ER and metabolic stress may also help to create immune suppressive environments through non-cell autonomous mechanisms, as we discussed earlier [212,221,224]. Nevertheless, this proposed mechanism is speculative and based on associative evidence, and further research is needed to directly address these important questions regarding aneuploidy and immune evasion in cancer. 

CIN may also help cells overcome immunodetection, although in many cases the exact mechanism is not clear. One study found CIN initially increased tumor cell immunogenicity, consistent with other reports [322,326], but continued evolution under immune selection promotes the proliferation of aneuploid cells that are able to suppress MHC class I antigen presentation and avoid immune detection [329]. A possible mechanism by which CIN and karyotype changes can mediate immune evasion is arm-level or focal deletions on chromosome 6 that result in human leukocyte antigen LOH, which was detected in about 40% of non-small cell lung cancers [330]. Cancer cells with human leukocyte antigen LOH produce less neoantigens and are less susceptible to immune predation, giving them a selective advantage in tumors [330]. Immune evasion, however, can also be achieved by karyotype-independent means. For example, epigenetic silencing of mutated genes (which can generate neoantigens and promote immune clearance of the cells harboring them) or of genes involved in the MHC-I antigen presentation pathway can allow cells to escape destruction by the immune system [329,331]. 

Immune pressure can dramatically influence clonal selection in tumors [332], leading to the dominance of less immunogenic sub-clones with favorable genomic and epigenetic alterations. Based on the apparent immunogenicity of aneuploid cells [322,323,326,329], it is tempting to speculate that the physiological consequences of aneuploidy, such as inflammation and the recruitment of immune cells, create a hostile immune predatory environment at first, but through ongoing genomic and environmental evolution a beneficial, immune suppressive TME and/or less immunogenic sub-clones emerge (Figure 3). Further work is needed to elucidate these dynamics through rigorous experimental studies.

#### 5.2.4. Increased Motility in Aneuploid and Polyploid Cells May Provide a Fitness Advantage in Some Contexts

Under certain conditions, motile phenotypes may be advantageous for cells. Mathematical modeling of tumors has shown that there is often a fitness trade-off between proliferation and motility (“go or grow” trade-off) and that it may be advantageous for a cell to be highly motile in certain conditions [333,334]. For example, in rapidly proliferating areas of a tumor, crowding and nutrient scarcity may make it advantageous for a cell to be able to escape such an environment. Thus, karyotypic changes that lead to increased motility could be selected for in or around these areas. Aneuploid cells have been found to be more invasive than diploid counterparts in a protein matrix meant to mimic the ECM [178]. Similarly, near-tetraploid cancer cells exhibited increased migratory and invasive behaviors compared to near-diploid cells [251]. Aneuploidy was also found to play a role in the phenotypic switch known as EMT. This phenotypic switch to the mesenchymal state leads to increased motility and is associated with metastasis [335]. During spontaneous transformation of mouse epithelial cells, aneuploidy arose concurrently with gene expression changes associated with EMT [336]. Another study found that EMT observed in cultured cells was associated with specific, recurrent changes in chromosome content, which affected the expression of ZEB1 and intercellular junction proteins central to the EMT process [337]. There is also *in vivo* evidence linking aneuploidy to EMT. Across 27 tumor types, the degree of aneuploidy positively correlated with the levels of EMT-related gene expression across 27 tumor types [257]. In addition to aneuploidy *per se*, it has been reported that chromosome missegregation can also induce EMT to promote invasive and metastatic phenotypes via cGAS-STING activation if micronuclei rupture [338].

#### 5.2.5. Effects of the TME on Karyotypically Abnormal Cells—Summary

Collectively, the findings discussed in this section demonstrate the principle that aneuploidy can provide cells with fitness advantages in certain contexts. Nonetheless, much remains to be uncovered about the interplay between aneuploidy and selective conditions in the complex contexts of tumors. Characteristic patterns of aneuploidy have been reported for different tumor types [339]. It has been proposed that these recurrent aneuploidies might enhance fitness by reinforcing the active transcriptional pathways specific to a given cell type [166,340]. It is also be possible, however, that these cancer-specific aneuploidy patterns are influenced by physiological differences in the tissue environment specific to the anatomical site. For example, a recent pan-cancer analysis of chromosome arm aneuploidies revealed that 7p gain and 10q loss—two recurrent events in primary brain tumors—were enriched in metastases to the brain relative to the primary site [300], suggesting tissue-specific environments may exert selective pressure that define the genomic evolution of tumors at their primary and metastatic sites. It will be important to design organoid and xenograft models to understand how these genome-environment relationships contribute to tumorigenesis.

## 6. Concluding Remarks

We have discussed how karyotype aberrations arise from cellular errors and environmental conditions; we have also explored the balance of forces that determines the extent of karyotype heterogeneity in a population, and the role of the bidirectional interaction between karyotypically abnormal cancer cells and the environment in shaping the TME and driving tumor evolution. While tremendous progress has been made in understanding how genomic and environmental alterations individually contribute to cancer, continued effort to integrate these fields has the potential to expand our knowledge of tumor progression. For instance, the role of niche construction in cancer is not well understood, and particularly the role of aneuploidy in niche construction has not been directly addressed to our knowledge. Therefore, many fundamental questions remain open. For instance, does the accumulation of aneuploidy in tumors exacerbate changes in the TME, diversifying tumor ecology across time and space? It seems plausible that the eco-evolutionary interactions that we discuss in this review act in tumors as a feedback loop that bolsters genomic and/or environmental heterogeneity, thereby driving tumor progression. How niche construction alters the spatial patterning of environmental niches and cell populations in tumors, and the consequences of this for disease progression and treatment response is unclear. Recognizing the parallels between species-environment dynamics in natural ecosystems, researchers have begun studying cancer from an ecological perspective and taking systems-level approaches. By integrating data from in vitro and in vivo systems, genomic and molecular analyses, bioinformatics, and mathematical modeling, we hope that these important questions can be answered. Indeed, experimental methods such as laser capture microdissection combined with single cell analyses (LCM-seq) are already being used to gain better understanding of spatial and functional relationships between different cells within a tumor and between cells and specific microenvironmental niches [341]. Such multimodal analyses integrating genomic, transcriptomic, epigenomic, and microenvironmental data are providing new insights into cancer biology [342,343]. 

Analysis of other complex systems has revealed various “leverage points” at which manipulation leads to amplified effects in the system [344]. Thus, experimental and mathematical analysis of niche construction and related ecological and evolutionary feedbacks in tumors may help to identify the processes central to cancer development, determine the best ways to disrupt the abnormal dynamics at play in cancerous tissue, and either return the system to a less malignant state or push the tumor to the point of collapse. Through a better understanding of the interactions and forces—genomic, environmental, and others—that shape tumor ecosystems, we hope that potent new therapeutic strategies will emerge.

## Figures and Tables

**Table 1:** The effects of karyotypically abnormal cells on the TME.

Experimental System	Cellular Effect(s)	Influence of the Cellular Effect(s) on the TME
Budding yeast [208]. HCT-116 and hTERT-immortalized RPE-1 cells with various trisomies and tetrasomies [206]. CENP-E inhibited HeLa cells [283].	Endoplasmic reticulum (ER) stress: Protein aggregates [208]. Compromised proteosome and chaperone proteins [206,208]. Impaired protein folding [206].	ER stress can transmit from cell to cell, including from cancer to stromal cells such as macrophages and dendritic cells [209,210,211]. ER stress in dendritic cells can lead to XBP1 activation, altered lipid homeostasis, and repressed T cell-dependent anti-tumor immunity [209]. Aneuploidy positively correlated with gene expression associated with ER stress and unfolded protein response (UPR) and negatively correlated with intra-tumor T cell cytolytic activity [212].
Mouse embryonic fibroblasts (MEFs) with Trisomy 1, 13, 16, or 19 [180]. Spindle assembly checkpoint (SAC) deficient MEFs [214]. Trisomic MEFs and chromosomally unstable cancer cell lines [219]. Haploid yeast strains with disomies for each chromosome [220]. HCT-116 and hTERT-immortalized RPE-1 cells with various trisomies and tetrasomies [215]. A near-tetraploid and a near-diploid line of Ehrlich’s ascites tumor [217].	Altered metabolism: Increased production of lactate, glutamate, and ammonium; increased glucose and glutamine consumption [180,214,219,220]. Altered nucleotide and membrane metabolism [215]. Altered consumption and production of various metabolites [219]. Increased glycolytic activity in near-tetraploid tumor cells compared to near-diploid tumor cells [217].	Increased lactate is a common cause of acidosis in tumors [284]. Increased lactate production may result in secretion of lactate into the tumor microenvironment. Increased glucose and glutamine consumption may result in their removal from the environment and other metabolic changes may also contribute to differences in the nutrient landscape observed in tumors [225,285]. Low pH in the extracellular environment may suppress anti-cancer immune response [286].
Spindle assembly checkpoint (SAC) deficient MEFs [214]. MEFs and human primary fibroblasts with downregulated BUB1 and SMC1A [182]. Aurora B inhibited U2OS and HCT-116 cells [287]. Budding yeast with various aneuploidies [208,228].	Altered reactive oxygen species (ROS) homeostasis and elevated ROS levels associated with aneuploidy and chromosomal instability [182,208,214,287].	Increased cellular ROS levels may translate to elevated tissue ROS levels, as observed in tumors [230]. Cancer cell-induced oxidative stress in cancer-associated fibroblasts leads to excessive production of lactate, ROS, and nitric oxide, which can be released in the TME and promote aneuploidy in adjacent cancer cells [235]. Oxidative stress can cause inflammation [236], which is a hallmark of cancer [26,237].
MEFs and human primary fibroblasts with downregulated BUB1 and SMC1A [182]. Nocodazole and Reversine treatment in HCT-116 and hTERT-immortalized RPE-1 cells [181]. Cancer cell lines with high levels of multipolar divisions [57]. DLD-1 and hTERT-immortalized RPE-1 p53^-/-^ cells undergoing multipolar divisions after induced cytokinesis failure [59].	CIN-associated cell death [57,59,182]. CIN-associated senescence [181,182].	Cell death can release stimulatory factors to promote proliferation of nearby cells [239,240]. Cell death can promote inflammation and immune cell recruitment [241,242]. The senescence-associated secretory phenotype (SASP) is associated with cell proliferation, inflammation, cell differentiation or phenotype switching, tissue remodeling, angiogenesis, and invasion [246,247]. Senescent cells can help neighboring cells escape immune detection by cleaving cell surface receptors in NK cells and potential target cells [248,249].
Various cell lines treated to induce cytokinesis failure, including DLD-1, HCT-116, MCF10A, and hTERT-immortalized RPE-1 and BJ fibroblast cells [57,59,171,266,268]. Aneuploid colorectal cancer cell lines compared to diploid ones [259].	Acquisition of extra centrosomes occurs with whole genome duplication (WGD) [57,59]. Note, other molecular changes may be required for cells to retain WGD-associated extra centrosomes, as they are quickly lost in cell culture [59]. Altered centrosome homeostasis proposed to occur due to aneuploidy (speculation and associational evidence) [259,261].	Centrosomal defects meant to mimic those seen in cancer disrupted tissue organization in 3D cultures [265]. Extra centrosomes and/or centrosomal defects can promote invasiveness in cells harboring them [266] and in adjacent cells [267]. Extra centrosomes have been linked to a secretory phenotype similar to SASP, known as the extra centrosome-associated secretory phenotype (ECASP) [267].
